# Discovery of Melittin as Triple-Action Agent: Broad-Spectrum Antibacterial, Anti-Biofilm, and Potential Anti-Quorum Sensing Activities

**DOI:** 10.3390/molecules29030558

**Published:** 2024-01-23

**Authors:** Hongyan Yang, Rong Ma, Jiarou Chen, Qian Xie, Wenhui Luo, Pinghua Sun, Zheng Liu, Jialiang Guo

**Affiliations:** 1 School of Medicine, Foshan University, Foshan 528000, Chinagaoagao@163.com (J.C.); xieqian@fosu.edu.cn (Q.X.); 2 College of Pharmacy, Jinan University, Guangzhou 510632, China; pinghuasunny@163.com; 3 Guangdong Yifang Pharmaceutical Co., Ltd., Foshan 528244, China; haoayhy@126.com

**Keywords:** antimicrobial peptides, melittin, antibacterial, anti-biofilm, anti-quorum sensing

## Abstract

The development of antibiotic-resistant microorganisms is a major global health concern. Recently, there has been an increasing interest in antimicrobial peptides as a therapeutic option. This study aimed to evaluate the triple-action (broad-spectrum antibacterial, anti-biofilm, and anti-quorum sensing activities) of melittin, a membrane-active peptide present in bee venom. The minimum inhibitory concentration and minimum bactericidal concentration of the melittin were determined using the microdilution method and agar plate counting. Growth curve analysis revealed that melittin showed a concentration-dependent antibacterial activity. Scanning electron microscope analysis revealed that melittin treatment altered the morphology. Confocal laser scanning microscope revealed that melittin increased the membrane permeability and intracellular ROS generation in bacteria, all of which contribute to bacterial cell death. In addition, the crystal violet (CV) assay was used to test the anti-biofilm activity. The CV assay demonstrated that melittin inhibited biofilm formation and eradicated mature biofilms. Biofilm formation mediated by quorum sensing (QS) plays a major role in this regard, so molecular docking and molecular dynamics analysis confirmed that melittin interacts with LasR receptors through hydrogen bonds, and further evaluates the anti-QS activity of melittin through the production of virulence factors (pyocyanin, elastase, and rhamnolipid), exopolysaccharides secretion, and bacterial motility, that may be the key to inhibiting the biofilm formation mechanism. The present findings highlight the promising role of melittin as a broad-spectrum antibacterial, anti-biofilm agent, and potential QS inhibitor, providing a new perspective and theoretical basis for the development of alternative antibiotics.

## 1. Introduction

The World Health Organization (WHO) claims that the indiscriminate use of antibiotics has increased bacterial resistance, resulting in the development of so-called “superbugs” that pose a threat to human health and have grown to be a challenging issue in the field of global health [1,2,3]. Bacterial biofilms are intricate three-dimensional structures covered in extracellular macromolecules and formed when bacteria adhere to the surface of living or inanimate things and are the primary contributor to bacterial resistance [4,5]. Biofilm formation is a highly complex process that is closely associated with quorum sensing (QS). QS refers to the ability of bacteria to detect and respond to bacterial population density through genetic regulation [6]. When a bacterial population reaches high densities, QS regulates a variety of activities in bacteria, including biofilm formation, antibiotic resistance, bioluminescence, and virulence factor production [7]. QS in *Pseudomonas aeruginosa* involves two key systems, LasI-LasR and RhlI-RhlR, which turn on virulence genes encoding exotoxin A, protease, and elastase, and exert an influence on the host immune system [8]. In the past decade, QS inhibitors (QSIs) have been considered to be agents that potentially can reduce pathogenicity and overcome bacterial antibiotic tolerance [9].

Antimicrobial peptides (AMPs) are extensively found in animals, plants, and numerous microbes, and play a crucial role in innate immune defense [10]. They consist of 20~60 amino acid residues and have a molecular weight of approximately 2000~7000 Da. They exhibit anti-inflammatory, anti-MRSA, and diabetes-fighting properties [11,12]. It is worth noting that AMPs are not susceptible to bacterial resistance due to their different targets, making them ideal candidates for antibacterial drugs [13,14]. Melittin is the primary bioactive component in European bee venom. It consists of 26 amino acids with a relative molecular mass of 2849 Da and a typical *α*-helical conformation with amphiphilic properties [15,16]. It has been attracting increasing attention due to its excellent antibacterial properties [17]. However, as far as we know, the mechanism of melittin on antibacterial and biofilm activity of different strains is not clear at present, and research on melittin on bacterial QS has rarely been reported.

In this study, melittin was utilized to explore the antimicrobial activity against bacteria. Initially, the minimum inhibitory concentration (MIC) and minimum bactericidal concentration (MBC) were determined. Then, we studied alterations in the morphology, membrane permeability, reactive oxygen species (ROS) generation, and biofilm inhibition and eradication activities to determine its mode of action against bacteria. Furthermore, we also explored the impact of melittin on bacterial motility, exopolysaccharide (EPS), and virulence factor production of *P. aeruginosa*. This study is significant for improving the treatment of *P. aeruginosa* clinical infections.

## 2. Results

### 2.1. Antimicrobial Activities of the Melittin In Vitro

To better evaluate the future potential application of melittin, the bacterial strains including six clinical isolates in addition to two standard strains were used. The results showed that melittin inhibited and killed all strains, with MIC values ranging from 4 to 64 μg/mL, and MBC values ranging from 8 to 128 μg/mL. Details and results are presented in Table 1. The inhibitory effect of melittin on *staphylococcus aureus* and *Acinetobacter baumannii* was remarkable, but a higher concentration of melittin was required to inhibit *Klebsiella pneumoniae*.

**Figure 1:**
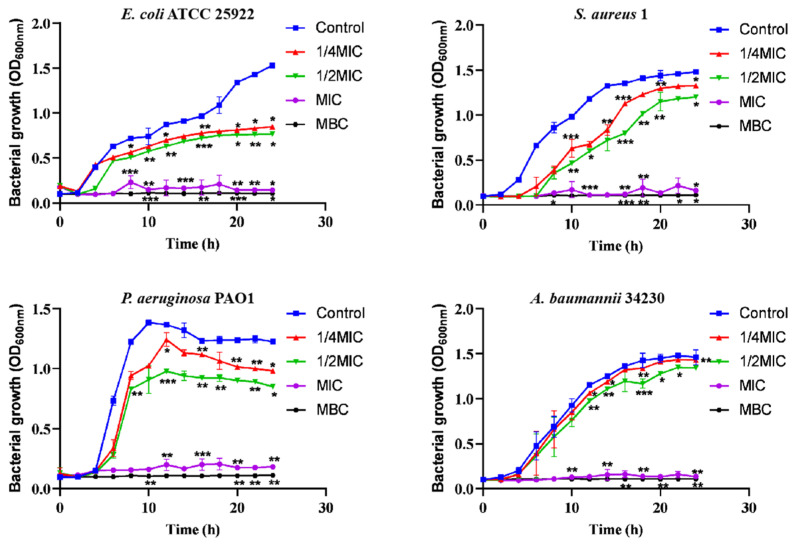
Bacterial growth curves at different melittin concentrations. Different concentrations of melittin inhibited the growth of *E. coli* ATCC 25922, *S. aureus* 1, *P. aeruginosa* PAO1, and *A. baumannii* 34230 at 1/4 MIC, 1/2 MIC, MIC, and MBC, respectively. Data are presented as mean ± standard deviation, *n* = 3. * *p* < 0.05, ** *p* < 0.01, *** *p* < 0.001 compared with control (0-melittin) group.

In addition, according to the recent annual report of the China Antimicrobial Surveillance Network (CHINET) (http://www.chinets.com/Data/AntibioticDrugFast) (accessed on 25 November 2022), China Bacterial Drug Resistance Surveillance Network, four representative strains were selected for further testing. After treatment with different concentrations of melittin (1/4 MIC, 1/2 MIC, MIC, MBC) for 2 h, melittin showed inhibitory effects against the four strains of bacteria (Figure 1), compared with the control (0-melittin) group. Bacteria in the absence of melittin grew the fastest and reached the highest plateau in the series. The bacterial growth rate at 1/2 MIC was slower than that at 1/4 MIC. The results indicated that the growth of bacteria was completely arrested during MIC treatment.

### 2.2. Effect of Melittin on Bacterial Cell Morphology

**Figure 2:**
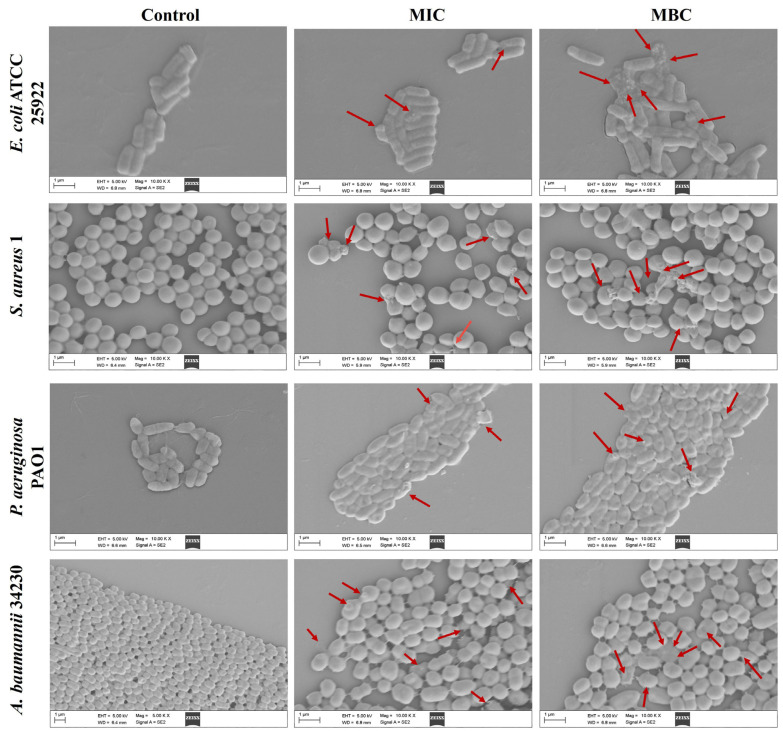
Effects of melittin on cell morphology at MIC and MBC. *E. coli* ATCC 25922, *S. aureus* 1, *P. aeruginosa* PAO1, *A. baumannii* 34230. PBS was used as the control. The red arrows indicate the site of the cell damage. The scale bars represent 1 µm.

Scanning electron microscope (SEM) analysis was performed to observe the effects of melittin on bacterial cells at MIC and MBC. As shown in Figure 2, after 24 h of exposure to MIC and MBC, the bacteria in the control group (PBS) without melittin remained intact, while the cell morphology under MIC concentration wrinkled, and broken, and the morphological changes were more severe at MBC. The results showed that bacterial exposure to both MIC and MBC led to cell damage.

### 2.3. Melittin on Bacterial Cell Membrane Permeability and Cell Death

**Figure 3:**
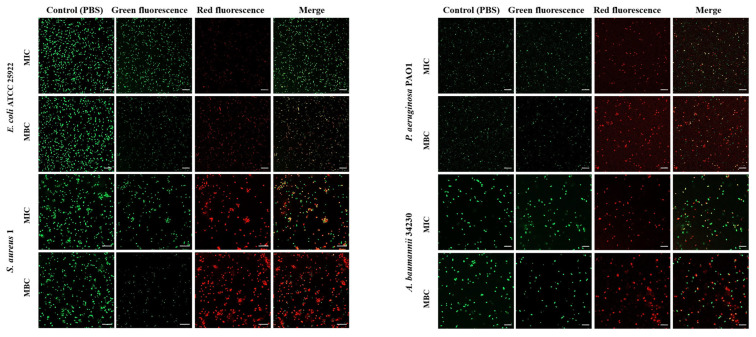
Effect of melittin at MIC and MBC on bacterial cell membrane permeability. *E. coli* ATCC 25922, *S. aureus* 1, *P. aeruginosa* PAO1, *A. baumannii* 34230, and PBS-treated bacteria were incubated for 10 h and stained with Propidium iodide (PI) and SYTO-9. The cells were observed through Confocal laser scanning microscope (CLSM) for red fluorescence (PI) and green fluorescence (SYTO-9) at excitation and emission. The scale bars represent 10 µm.

To investigate whether melittin can kill bacteria by breaking the cell membrane, propidium iodide (PI) and SYTO 9 staining were performed after melittin treatment, and PBS treatment was used as a control. SYTO 9 can penetrate the cell membrane of Gram-positive bacteria, Gram-negative bacteria, and living and dead cells and releases green fluorescence. PI cannot penetrate intact cells but can produce red fluorescence after cell membrane destruction. As shown in Figure 3, the control group did not show red fluorescence, indicating that melittin changed the permeability of the cell membrane of *E. coli* ATCC 25922, *S. aureus* 1, *P. aeruginosa* PAO1, and *A. baumannii* 34230. In addition, the red fluorescence at MBC concentration was greater than that at MIC concentration, indicating that the change in membrane permeability of the four strains gradually increased by increasing the melittin concentration (Table S1). Among these, the cell membrane of *S*. *aureus* 1 underwent the most significant permeability change (Figure 3).

### 2.4. ROS Generation in Bacterial upon Exposure to Melittin

**Figure 4:**
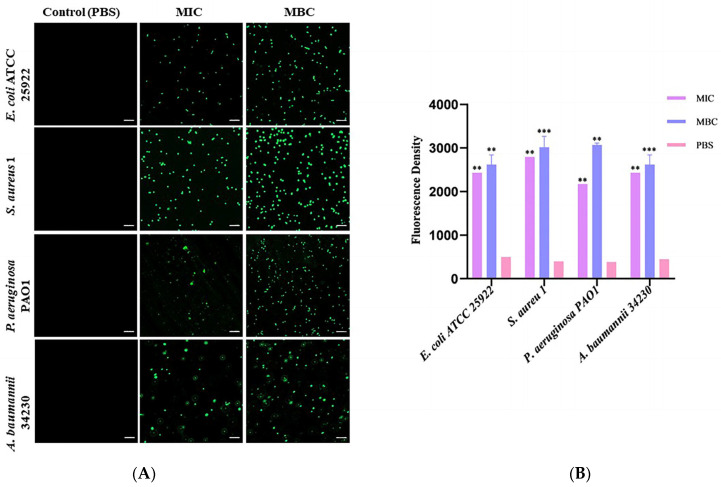
Effects of melittin at MIC and MBC on reactive oxygen species (ROS) production in bacterial cells. (**A**) Fluorescence of ROS of four bacteria under laser confocal observation (*E. coli* ATCC 25922, *S*. *aureus* 1, *P*. *aeruginosa* PAO1, and *A. baumannii* 34230). The scale bars represent 10 µm. (**B**) Quantitative analysis of the intracellular ROS fluorescence intensity. PBS was used as the control. ** *p* < 0.01, and *** *p* < 0.001 compared with PBS-treated group.

To determine whether the bactericidal mechanism of melittin is related to intracellular ROS levels, 2′,7′-dichlorofluorescein diacetate (DCFH-DA) staining was used to detect the changes in intracellular ROS levels in melittin-treated and non-treated bacteria. As shown in Figure 4, compared with the PBS-treated group, the fluorescence intensity of ROS in the MBC concentration treatment group was stronger than that in the MIC treatment group. With an increase in peptide concentration, ROS levels in the cells of the four bacteria gradually increased in a concentration-dependent manner. Notably, melittin induced ROS with the greatest intensity in *S*. *aureus* 1 (Table S2). Therefore, the bactericidal mechanism against these four bacteria was closely related to the accumulation of ROS.

### 2.5. The Ability of Biofilm Formation

Bacterial biofilms are highly resistant to antibiotics and host immune defense mechanisms [18,19]. The biofilm-forming capacity of clinical and standard strains was measured using crystal violet (CV) staining. The results showed that all isolates formed biofilms of varying degrees (Table 2). The maximum and minimum OD values of the clinical isolates were 3.883 and 0.248, respectively. The maximum and minimum OD values of ATCC strains were 3.208 and 1.849, respectively. Based on these results, bacteria were classified as strong, moderate, or non-biofilm producers.

### 2.6. Inhibition and Eradication of Biofilm by Melittin

**Figure 5:**
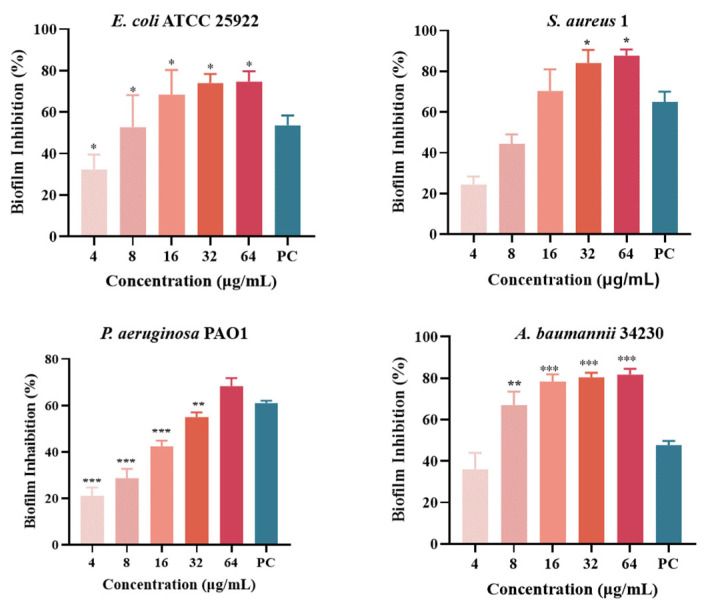
Biofilm inhibition of melittin against *E. coli* ATCC 25922, *S. aureus* 1, *P. aeruginosa* PAO1, and *A. baumannii* 34230. Compared to the positive control group (PC = positive control, azithromycin), data are shown as mean ± SD, and individual biological replicates are shown (*n* = 3); * *p* < 0.05, ** *p* < 0.01, *** *p* < 0.001 compared with PC group.

**Figure 6:**
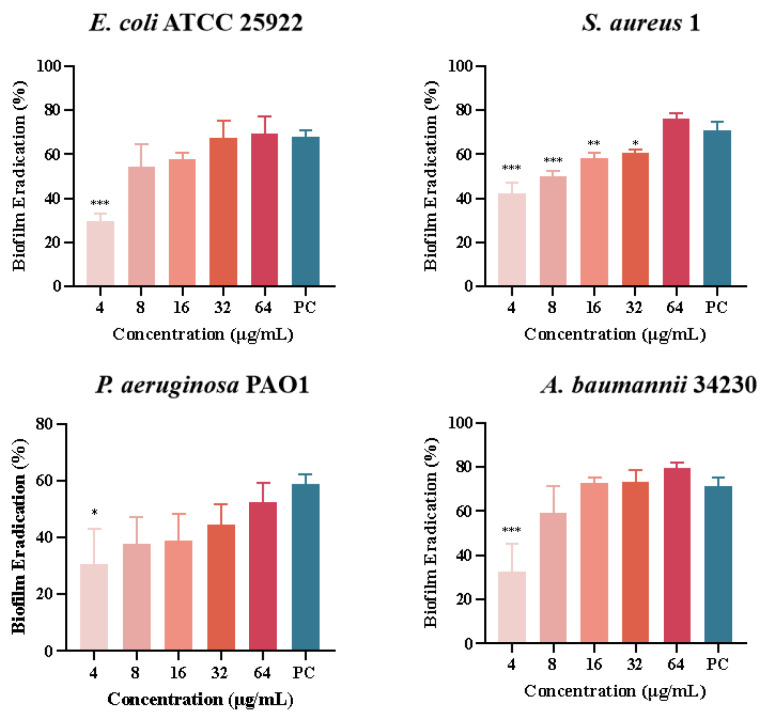
Biofilm eradication of melittin against *E. coli* ATCC 25922, *S. aureus* 1, *P. aeruginosa* PAO1, and *A. baumannii* 34230. Compared to the positive control group (PC = positive control, azithromycin), data are shown as mean ± SD, and individual biological replicates are shown (*n* = 3); * *p* < 0.05, ** *p* < 0.01, *** *p* < 0.001 compared with PC group.

Quantitative analysis of biofilm formation inhibition and eradication after melittin treatment was performed using CV staining, as shown in Figure 5. The results showed that the inhibition rates of melittin against *E*. *coli* ATCC 25922, *S*. *aureus 1*, *P*. *aeruginosa* PAO1, and *A*. *baumannii* 34230 ranged from 20% to 87%. In addition, *S*. *aureus* 1 and *A*. *baumannii* 34230 showed better biofilm inhibition at 64 μg/mL with 87% and 85%, respectively. These results showed that melittin had a concentration-dependent inhibitory effect against all four strains, compared with the positive control group. The results showed that melittin could eradicate the biofilms of the four bacteria. Compared with the positive control (72%), melittin had the best removal effect (80%) against *A. baumannii* 34230. These results also demonstrated a concentration-dependent biofilm eradication effect of melittin (Figure 6).

### 2.7. Molecular Docking and Dynamic Simulation

**Figure 7:**
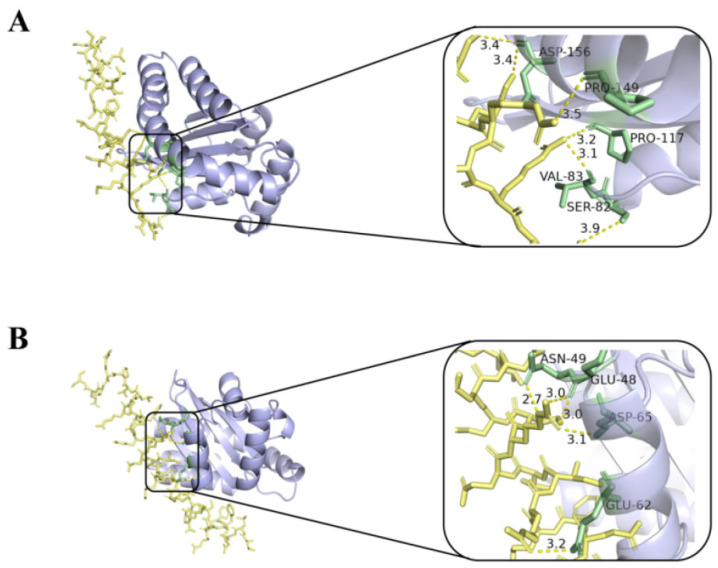
Molecular docking 3D image of melittin (**A**), LL-37 (**B**), and LasR protein model. Blue represents the receptor LasR, yellow represents the polypeptide ligand, and green represents the amino acid residues of the polypeptide-protein interaction.

The interaction of melittin and LL-37 peptide with the receptor LasR active site was studied by molecular docking analysis. In Figure 7, melittin interacts with residues of ASP-156, PRO-149, PRO-117, VAL-83, and SER-82 of the LasR protein via the H-bond (yellow dashed line) (Figure 7A). In addition, control LL-37 was found to interact with ASN-49, GLU-48, ASP-65, and GLU-62 via H-bonds (Figure 7B).

**Figure 8:**
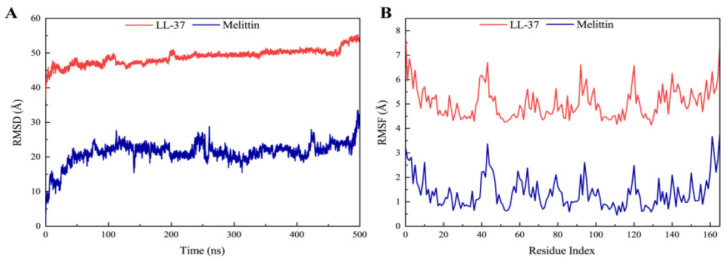
(**A**) RMSD profile and (**B**) RMSF profile of LasR-Melittin and LasR-LL-37 during molecular dynamics simulations at 500 ns.

In protein–ligand complexes, peaks represent protein changes during the simulation. To further investigate the conformational change of the receptor protein in the presence of melittin, molecular dynamics simulations at 500 ns were performed, and the stability of the complex was assessed by root–mean–square deviation (RMSD) and root–mean–square fluctuation (RMSF) values. As shown in Figure 8A, the LasR-melittin complex was found to have negligible or low fluctuations (Table S3), indicating that its conformation was relatively stable over this period. However, the RMSD value of the LasR-LL-37 complex showed to be relatively stable throughout the simulation (Table S3). In addition, RMSF indicates the position deviation of the amino acid residue with respect to the reference position. In Figure 8B, RMSF values of protein–ligand complexes were all less than 3.5 Å (Table S4) during the simulation period. Notably, the LasR-melittin complex and the LasR-LL-37 complex in the initial and final RMSDs during the whole simulation period were not found to a significance difference. This showed a stable binding of melittin with LasR.

### 2.8. The Influence of EPS

**Figure 9:**
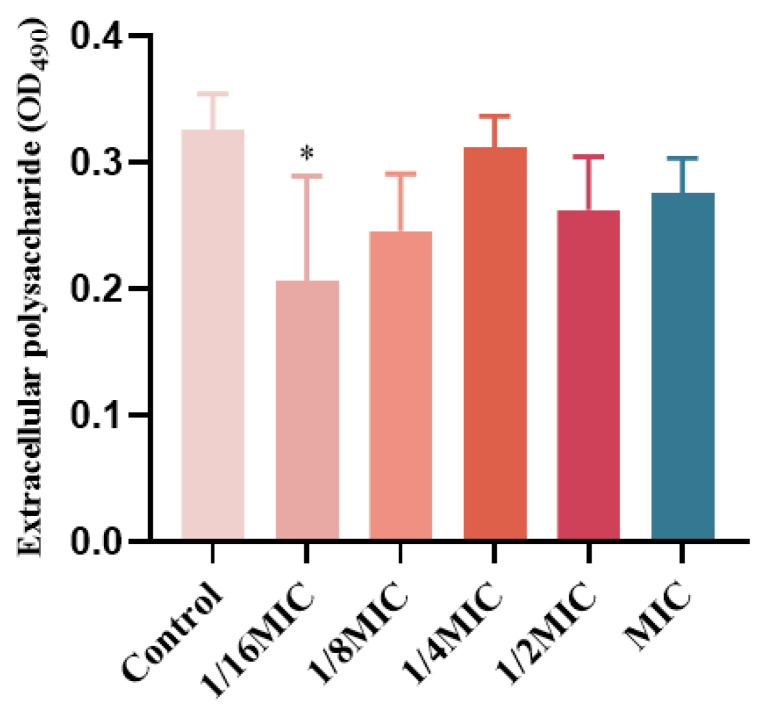
Effect of melittin on EPS in *P. aeruginosa* PAO1 biofilms. Data are shown as the mean ± SD, and individual biological replicates are shown (*n* = 3); * *p* < 0.05 compared with control (0-melittin) group.

EPS in biofilms are a class of carbohydrate compounds synthesized and secreted by extracellular microorganisms, composed of nucleic acids, polysaccharides, lipids, and proteins, and act as a diffusion barrier to antimicrobial agents [20,21]. *P*. *aeruginosa* matrix is derived from secreted proteins and lysed cells [22,23]. Therefore, the effect of melittin on the EPS content in *P*. *aeruginosa* biofilms was determined. EPS production was inhibited at concentrations below the MIC (Figure 9), compared with the control (0-melittin) group. The result indicated that melittin could affect the formation of EPS in *P. aeruginosa* PAO1.

### 2.9. Effect of Melittin on the Production of Virulence Factors

**Figure 10:**
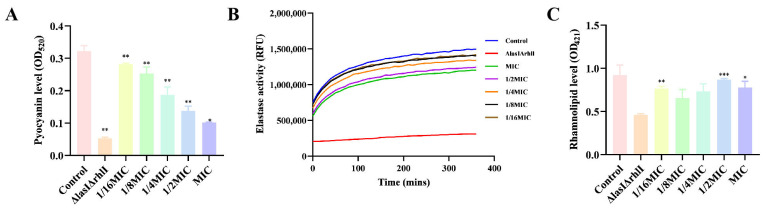
Effects of different concentrations of melittin on virulence factors. (**A**) pyocyanin, (**B**) elastase, and (**C**) rhamnolipid production. *n* = 3. * *p* < 0.05, ** *p* < 0.01, *** *p* < 0.001 compared with control (0-melittin) group.

Bacterial biofilms are also regulated by strong cell-to-cell communication called QS. It is a cellular communication mechanism that involves inducers and regulators. In *P. aeruginosa* PAO1, complex signaling molecules increase the severity of infection in the host [24,25,26]. To this end, melittin was used to determine relevant virulence factors secreted by the QS system in *P*. *aeruginosa* PAO1. The results showed that melittin (1/16 MIC, 1/8 MIC, 1/4 MIC, 1/2 MIC, MIC) affected the production of pyocyanin, elastin, and rhamnolipid in *P*. *aeruginosa* PAO1, compared with Δ*lasI*Δ*rhlI* mutant strain (Figure 10). Melittin has strong QS inhibitory activity against PAO1, which can be a new means of controlling bacterial biofilms. Taken together, melittin is expected to serve as a potential new strategy for biofilm inhibitors in the treatment of infections.

### 2.10. Effects on Swarming, Swimming, and Twitching Motility

**Figure 11:**
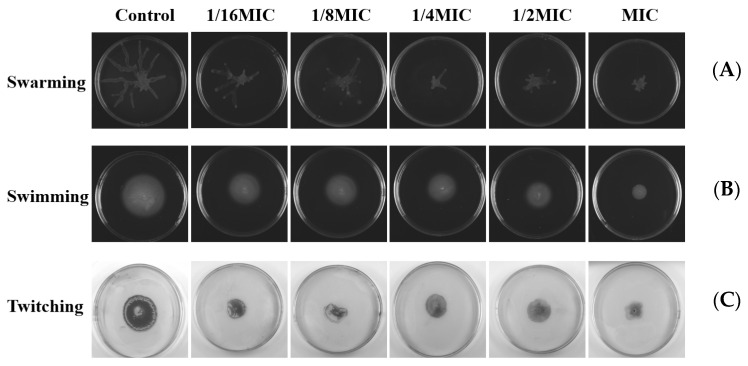
Effects of different concentrations of melittin on swarming, swimming, and twitching motility. (**A**) Swarming motility, (**B**) swimming motility, and (**C**) twitching motility. The results shown are representative of the results of three independent experiments.

Swarming, swimming, and twitching control the formation of bacterial biofilms through attachment and complex signal transduction systems. Then, we investigated the influence of melittin on these movements. The double-deletion strain PAO1-Δ*lasI*Δ*rhlI* was used as the negative control. As shown in Figure 11, compared with the negative control group, swarming, swimming, and twitching motilities of *P. aeruginosa* PAO1 were inhibited after melittin treatment.

## 3. Discussion

Microbial cells exist in a substantial number of environmental niches, so biofilms are ubiquitous and can be found in a wide range of environments, including water pipes, medical devices, food processing equipment, human tissues, and even human organs [27,28,29,30]. Once a bacterial infection has become established, it is very difficult to eradicate. Their biofilms create highly impenetrable barriers to host immune defenses and antibiotics, which can result in the emergence of resistant strains and alarming morbidity and mortality [31,32]. In this situation, the search for and develop new antibacterial compounds is demanded to prevent the formation of biofilm and to strengthen the clinical treatment of bacterial infections.

AMPs are the first line of defense that forms innate immunity and protects the host from invading pathogens. AMPs have multiple mechanisms of action, and their targeting of a broad spectrum of pathogens and not easy induction of resistance have attracted much attention from researchers around the world. Melittin, one of the AMPs, shows excellent antimicrobial activity. However, whether the mechanism of melittin is related to QS requires further investigation. Therefore, the underlying mechanisms of antibacterial, anti-biofilm, and QSI activities of melittin were investigated in this study. The antibacterial effect of melittin against standard and clinical strains (*E. coli*, *S. aureus*, *P. aeruginosa*, *A. baumannii*, and *K. pneumoniae*) was determined in vitro. The results showed melittin had antibacterial activities against these tested strains, particularly, melittin showed high activity against *S. aureus* and *A. baumannii* (Table 1). It is worth noting that melittin has antibacterial activity against Gram-positive and Gram-negative bacteria, which again proves that melittin has a broad spectrum of antibacterial effects as previously reported [33,34]. *E. coli*, *S. aureus*, *P. aeruginosa*, and *A. baumannii* are particularly important in human and veterinary medicine due to their pathogenicity, virulence, distribution, diversity of clinical signs, and problems related to public health. Thus, *E. coli* ATCC 24922, *S. aureus* 1, *P. aeruginosa* PAO1, and *A. baumannii* 34230 were selected to further study the antibacterial mechanism. The growth curves assay confirmed the concentration-dependent bactericidal action of melittin against bacteria. Moreover, the concentrations of melittin at MIC and MBC caused bacterial shrinkage and damage to bacterial morphology, and the permeability of the cell membrane was also changed (Figure 2 and Figure 3). Oxidative stress is cytotoxic and can induce apoptosis or necrosis of cells. To determine whether the bactericidal mechanism of melittin against bacteria is related to ROS production, the ROS level of bacteria was measured by confocal laser scanning microscope (CLSM) analysis. The results showed that melittin may induce an increase in ROS and thus play bactericidal roles (Figure 4). The unique three-dimensional structure of biofilms hinders the permeability of antimicrobial drugs, leading to a high level of resistance to antibiotics and host immune defenses [35]. The effect of melittin was investigated against bacterial biofilms using the CV staining method. The results showed that all eight strains could produce different degrees of biofilm, and melittin was able to significantly inhibit the biofilm formation of four strains (*E. coli* ATCC 24922, *S. aureus* 1, *P. aeruginosa* PAO1, and *A. baumannii* 34230) and eradicate mature biofilm in a concentration-dependent manner (Figure 5 and Figure 6).

*P. aeruginosa*, one of the ESKAPE pathogens, with multi-drug resistant strains, has been identified by the WHO as a key priority pathogen in need of new antimicrobial agents. The QS system of *P. aeruginosa* is crucial in coordinating multiple activities such as the formation of biofilms and the release of virulence factors [36]. LasI/LasR, RhlI/RhlR, and PqsA/PqsR are three major QS regulatory systems organized in *P. aeruginosa* in a hierarchical manner, which together regulate the production of virulence factors, where the LasI/LasR system is at the top of the hierarchy and is associated with bacterial motility [37,38]. Screening QSI by targeting the transcriptional regulatory factor LasR in the QS system of *P. aeruginosa* is a rapid and promising approach. Here, we also investigated whether melittin can be used as QSI for *P. aeruginosa* [38]. First, the anti-QS mechanism was analyzed by computer. The interaction between melittin and receptor protein was studied using molecular docking. H-bonds can possess the capability to sustain the stability of intricate molecules and play a pivotal role in molecular recognition. The findings revealed that both melittin and LL-37 could form stable complexes by hydrogen bonding to the active amino acid residues in the cavity structure of the LasR receptor protein (Figure 7). LasR formed more hydrogen interactions with melittin than with LL-37. To evaluate the stability of melittin and LL-37 in their respective binding bags, RMSF and RMSD curves showed that the LasR-melittin complex exhibited consistency with the LasR-LL-37 complex stability throughout the simulation (Figure 8). These results suggest that melittin may have the potential to inhibit the production of virulence factors by regulating QS. Pyocyanin, elastase, and rhamnolipid are representative virulence factors of *P. aeruginosa*, while EPS is also a significant component of the biofilm, occupying between 65% and 95% of the biofilm volume. EPS can promote adhesion and accumulation of bacteria, provide mechanical stability for extracellular polymeric substances of biofilm, and provide a barrier to host immune cells [39]. The results showed that melittin reduced the production of EPS (Figure 9). Compared to the control group, the expressions of pyocyanin, elastase, and rhamnolipid were significantly inhibited (Figure 10). In addition, the initial attachment of biofilm formation was closely related to the movement of bacteria, and swimming, swarming, and twitching movements were inhibited compared to the control group (Figure 11). Virulence factor secretion is the key factor in the pathogenicity of *P. aeruginosa*. The reduction of virulence factors not only indicates a decrease in the pathogenicity but also indicates an inhibition of the QS system, ultimately leading to a reduction in biofilm formation.

Rangel Karyne et al. [34] reported that melittin has a destructive effect on the cell membrane of *A. baumannii*, and also confirmed its antibacterial effect. However, current research on the effect of melittin on ROS production and QS in biofilm formation is still insufficient. Here, the inhibitory virulence factors production of melittin in QS was reported for the first time, and the antibacterial and anti-biofilm mechanisms of melittin were revealed. This provides theoretical guidance and reference significance for subsequent research on biofilms and QS. Additionally, the study showed that melittin has certain effects against both standard and clinical strains, and has certain inhibitory and destructive effects on biofilms, suggesting that melittin has potential applications in medicine, agriculture, and forestry in the future. Other studies have shown that melittin can inhibit the growth of *S. aureus*, *E. coli*, and *P. aeruginosa* in milk as well as inhibit biofilm formation, further confirming the application value of melittin in food [40].

**Figure 12:**
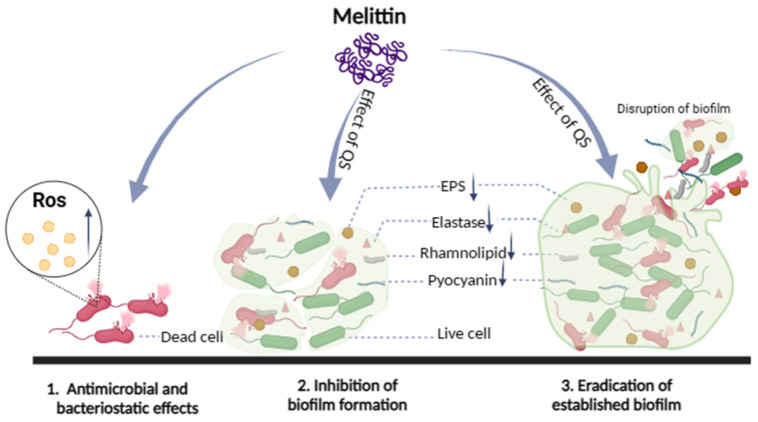
Schematic illustration of the multiple actions of melittin against bacteria.

To sum all, in the process of interaction between melittin and bacteria, melittin mainly destroyed the morphology of bacterial cells, changed the permeability of cell membranes, induced the proliferation of ROS in bacterial cells, and eventually resulted in cell death. Melittin inhibited biofilm formation and removed mature biofilm to exert anti-biofilm activity. Furthermore, melittin reduced the production of EPS and virulence factors (pyocyanin, elastase, rhamnolipid), and inhibited the movement of bacteria to exert potential anti-QS activity (Figure 12). Based on the above study of melittin interacting with bacteria, the mechanism of antibacterial and anti-biofilm activity of melittin important discoveries have been made. However, in the complex biological membrane structure, the effect of drugs on bacteria along with the change of the external environment, and many factors lead to bacterial tolerance, especially the combination of antibiotics and matrix components, anaerobic environment, and the presence of continuous cells. Further study on the molecular mechanisms underlying biofilm regulation is required to gain a better understanding of the molecular basis of biofilms, which will help expand the theoretical knowledge on the regulation of biofilm formation, develop new strategies for biofilm elimination, and extend their use to other fields.

## 4. Material and Methods

### 4.1. The Source of Melittin

Melittin was synthesized by Aladdin Biochemical Technology Co., Ltd. (Shanghai, China). Its purity exceeded 95%.

### 4.2. Bacterial Strains and Culture Conditions

The bacteria strains, including *E. coli* ATCC 25922, *S. aureus* ATCC 25923, *S. aureus* 1, *S. aureus* 3, *P. aeruginosa* PAO1, *A. baumannii* 34230, *A. baumannii* 57190, and *K. pneumoniae* 53735, were obtained from the U.S. Microbial Strain Conservation Center, Guangzhou Overseas Chinese Hospital, and Foshan Stomatological Hospital.

All bacterial colonies were inoculated in Luria broth (LB) (Solarbio, Shanghai, China) and incubated at 37 °C and 150 rpm. The 96-well microtitration plates were supplied by Corning Costar (New York, NY, USA).

### 4.3. Antimicrobial Activity Assays

The antibacterial activity of melittin was determined by measuring their MIC using the broth microdilution method according to the Clinical and Laboratory Standards Institute (CLSI) guidelines [41,42]. For MIC, a broth dilution susceptibility test was performed using Mueller–Hinton broth (MHB) [33,43,44]. Fresh bacterial colonies were incubated in MHB at 37 °C and 200 rpm overnight, a bacterial suspension with optical density (OD_600_) between 0.9 and 1 is equal to 10^9^ colony forming units (CFU)/mL, and the bacterial suspension was diluted with MHB broth to 10^6^ CFU/mL. In addition, 100 μL melittin (128~0.5 μg/mL) was simultaneously prepared with MHB in 96-well microplates. Each well contained a total of 200 μL of melittin and bacteria, and the microplates were incubated at 37 °C overnight. The tested concentration at which growth was observed to be completely inhibited was regarded as the MIC. For MBC, with reference to the determination method of MIC, 100 μL of liquid was taken from each well of the 96-well plate and evenly smeared with a smearing stick-on MH agar (MHA). The number of colonies was counted on the second day after 24 h of incubation at 37 °C. The lowest concentration without colony growth was taken as MBC of melittin against bacteria. Each test was measured in triplicate.

### 4.4. Growth Curve Assay

Bacteria were grown in 5 mL Tryptic Soy Broth (TSB) at 37 °C. The bacterial suspension of 10^6^ CFU/mL was collected, and the bacterial culture was divided into four flasks (250 mL), and 1/4 MIC, 1/2 MIC, MIC, and MBC of melittin were added, and the negative control group without melittin was used. The flask was incubated at 37 °C with constant oscillation (200 rpm), and bacterial growth was determined by measuring the absorbance of the culture at OD_600_ at two-hour intervals after treatment [45,46].

### 4.5. Effect of Melittin on Morphological Changes

SEM was used to observe the effect of melittin on bacteria morphology changes [47]. In brief, overnight culture bacteria were diluted to OD_600_ ≈ 0.05 with LB medium. Then, bacterial suspensions were treated with different concentrations of melittin (MIC and MBC), and PBS was used as a negative control. After incubation at 37 °C for 24 h, bacterial strains were harvested by centrifuging at 5000 rpm for 10 min and washed three times with sterile PBS. Subsequently, the bacteria were fixed with 2.5% (*v*/*v*) glutaraldehyde overnight at 4 °C. The samples were dehydrated in an ethanol gradient (30%, 50%, 70% 90%, and 100%) for approximately 10 min. The dehydrated bacterial strain suspension was then dropped onto a silicon wafer and dried. After the platinum coating, the samples were observed by SEM.

### 4.6. Live and Dead Cell Staining

To further determine the changes in cell membrane permeability, in this study, SYTO 9 was co-stained with PI to determine the effect of melittin on bacterial cell membrane permeability [48]. Bacterial suspensions (1 × 10^8^ CFU/mL) were incubated with the MIC and MBC of melittin, and PBS (negative control) at 37 °C for 10 h. Next, 1 mM PI and 10 mM SYTO 9 were added to the cells and incubated for 30 min in the dark. The cells were pelleted after centrifuging at 3500 rpm for 5 min, washed three times with PBS, and resuspended in PBS. Fluorescent images were acquired using a Zeiss LSM 800 confocal microscope (Oberkochen, Germany).

### 4.7. ROS Test

Intracellular ROS production was measured using DCFHDA with reference to the method described in the literature [49,50]. Bacteria were grown overnight in 5 mL LB medium to a final optical density of 10^8^ CFU/mL, incubated with different concentrations of melittin (the final concentration was MIC and MBC), saline solution was applied as negative control, and incubated at 37 °C for 10 h. The cells were pelleted after centrifugation at 3500 rpm for 5 min, washed three times with PBS, and resuspended in PBS. DCFH-DA was added according to the instructions for staining. The final concentration was 10 µmol/L, and the cells were incubated for 30 min in the dark. After washing and resuspension in PBS, the cells were observed under CLSM for the detection of green fluorescence at an excitation wavelength of 485 nm and an emission wavelength of 528 nm.

### 4.8. The Ability of Biofilm Formation

For the biofilm formation assay, bacteria were cultured in 5 mL TSB overnight at 37 °C. 150 μL bacteria prepared with ABTGC medium (0.1% CaCl_2_, 0.1% MgCl_2_, 0.1% FeCl_3_, 10% A10, 0.2% casamino acids, and 0.2% glucose) to a final OD_600_ of 0.01 were added to each well of 96 Well microplates (Costar, Corning, NY, USA) and incubated at 37 °C for 24 h. The wells were washed three times with PBS after incubation and air-dried. Subsequently, 150 μL anhydrous methanol was added to each well for biofilm fixation. After 15 min, the solution was removed, and the plates were allowed to dry at room temperature. The wells were stained with 150 μL of CV for 15 min, the solution was aspirated, and the wells were washed with tap water. Finally, 150 μL glacial acetic acid was added to each well, shaken for 5 min, and the absorption was measured at 595 nm. All experiments were repeated thrice [51,52].

The interpretation of biofilm formation ability was based on previous studies [53]. The optical density cut-off value (ODc) was established as three standard deviations (SD) above the mean of the optical density (OD) of the negative control as shown in the following formula: ODc = average OD of negative control + (3 × SD of negative control). The results were divided into the four following categories according to their optical densities as (1) strong biofilm producer (4 × ODc < OD); (2) medium biofilm producer (2 × ODc < OD ≤ 4 × ODc); (3) weak biofilm producer (ODc < OD ≤ 2 × ODc); (4) non-biofilm (OD ≤ ODc).

### 4.9. Effect of Melittin on Bacterial Strains Biofilm Inhibition and Eradication

The inhibitory and eradication ability of melittin against biofilm was determined by referring to the above CV method. For the biofilm formation inhibition assay, bacterial suspension (1 × 10^6^ CFU/mL) was added to a 96-well plate with melittin at different final concentrations (1/16 MIC, 1/8 MIC, 1/4 MIC, 1/2 MIC, and MIC) at 37 °C for 24 h, azithromycin (50 μg/mL) was applied as the positive control [52,54]. For the biofilm eradication assay, bacterial suspension (1 × 10^6^ CFU/mL) was added to 96-well plates (100 μL/well) and incubated at 37 °C for 24 h until biofilm formation. After incubation, the floating bacteria were washed with PBS. Each well was treated with melittin (0~64 μg/mL) and azithromycin (50 μg/mL). The plates were then incubated at 37 °C for 18 h. Quantitative inhibition and eradication of biofilm were performed using the CV method [55]. All antibiofilm experiments were assessed in triplicate.

### 4.10. Molecular Docking Analysis

The three-dimensional structures of the LasR receptor (PDB ID: 2UV0), LL-37 ligand (PDB ID: 2K6O), and the melittin ligand (PDB ID: 8AHIS) were obtained from the PDB database (https://www.rcsb.org) (accessed on 10 December 2022) for further computer simulation studies [56,57]. LasR Protein and melittin were pretreated using the Protein Prep Wizard in the Maestro system, and protein–polypeptide docking was performed using the HDOCK program (http://hdock.phys.hust.edu.cn/) (accessed on 10 December 2022) [58].

### 4.11. Molecular Dynamics Simulation Analysis

Molecular dynamics simulations were carried out to analyze the stability of LasR receptor and melittin ligand using the academic version. Firstly, the SPC water model was used to construct a dynamic system by dissolving the complex in a cube box filled with water, and the appropriate amount of Na^+^ ions and Cl^−^ ions was added to neutralize the charge of the system. An additional 0.15 M concentration was added to simulate the real protein environment. Based on the OPLS2005 force field, 500 ps energy minimization was performed. The system was completely relaxed before the formal molecular dynamics simulation, and the MD full (molecular dynamics simulation) was run for 500 ns at 300 K and 1 atm pressure. Except for the above parameters, the default settings were used [59,60].

### 4.12. EPS Assay

The EPS content was determined using the phenol–sulfuric acid method [61]. Single colonies of *P. aeruginosa* were picked and incubated overnight in LB to log phase and then diluted to OD_600_ = 0.01. Different concentrations of melittin (1/16 MIC, 1/8 MIC, 1/4 MIC, 1/2 MIC, MIC) and bacterial suspension were added into 96-well plates and incubated in incubators at 37 °C for 24 h. The suspension was washed 3 times with PBS, followed added saline solution ultrasound for 5 min, and then heated in an 80 °C water bath for 30 min. The supernatant was obtained by centrifugation at 10,000 rmp for 10 min. Subsequently, 100 μL supernatant was added into the test tube, 5% phenol and 500 μL concentrated sulfuric acid were added, and the absorption value at 490 nm wavelength was determined by ultraviolet spectrophotometer.

### 4.13. Pyocyanin Assay

Referring to the methods mentioned in the literature [62], the Δ*lasI*Δ*rhlI* mutant that showed the deficient ability to produce rhamnolipid and elastase was used as a positive control. A single colony of the test organism was selected and incubated overnight in 5 mL LB medium at a final OD_600_ = 0.01. Melittin (MIC, 1/2 MIC, 1/4 MIC, 1/8 MIC, and 1/16 MIC) was added and grown for 24 h at 37 °C (200 rpm). The supernatant was obtained by centrifuging the experimental culture at 10,000 rpm for 10 min. Then, 4 mL of the supernatant was transferred to a new test tube, and 1 mL of trichloromethane was added for complete mixing and extraction. After that, pyocyanin was extracted with 1 mL 0.2 M hydrochloric acid, and the absorbance was determined at OD_520_ [63].

### 4.14. Elastase Assay and Rhamnolipid Assay

A single colony of the test organism was selected and incubated overnight in 5 mL LB medium at a final OD_600_ = 0.01. Different concentrations of melittin (MIC, 1/2 MIC, 1/4 MIC, 1/8 MIC, and 1/16 MIC) were added and the cells were cultured at 37 °C (200 rpm) for 24 h. The supernatant was collected by centrifugation at 10,000 rpm for 10 min. For elastase, the filtered supernatant was later taken and assayed using an Elastase Assay Kit (Invitrogen, Waltham, MA, USA). For the rhamnolipid assay, the supernatant was extracted with ether and centrifuged at 10,000 rpm and 4 °C for 10 min. Afterward, the supernatant was transferred to a new centrifuge tube to extract the concentrated organic fraction, and deionized water and moss black phenol were added and incubated at 80 °C for 30 min. After cooling, the absorbance was measured at 421 nm [64,65]. The test was performed in triplicates.

### 4.15. Swarming, Swimming, and Twitching Motility Assay

Referring to the detection method described in the literature [66], the PAO1 strain was cultured in 5 mL LB overnight at 37 °C. The plates were prepared with agar (0.5%) and glucose (0.5%) and were cured at room temperature for 40 min. Then, 1 µL of PAO1 was added to the center of the plate and incubated upright at 37 °C for 16 h under controlled humidity. Plates were observed using a standard computer scanner.

### 4.16. Data Analysis

All experimental data were analyzed using GraphPad Prism software version 5 (GraphPad Software Inc., La Jolla, CA, USA). One-way analysis of variance (ANOVA) was performed to determine the statistically significant (*p* < 0.05) differences between the control and treatment groups for growth curve assay, ROS determination, biofilm inhibition, biofilm eradication, EPS, and virulence factor measurements. Each experiment was performed at least three times, and the data are expressed as mean ± SD.

## 5. Conclusions

In summary, melittin exhibited excellent performance against standard and clinical strains. Moreover, melittin could inhibit the initial biofilm formation and eliminate the preformed biofilm as evidenced by CV staining. Interestingly, even at or below MIC, melittin reduces EPS production by interfering with the bacterial QS system, reducing the release of virulence factors, and inhibiting bacterial movement, which leads to cell death. Therefore, due to its “triple-action”, melittin could be a promising alternative to antibiotics as a multi-antimicrobial agent, anti-biofilm agent, and QS inhibitor. The applicability of the bioactive melittin is expected to provide new methods for bacterial inhibition and control, which could be applied in different fields, such as agriculture, food processing, pharmaceutical, and healthcare industries, and can be developed as a new antibacterial drug.

## Figures and Tables

**Table 1:** MIC and MBC of melittin.

Strain	MIC (μg/mL)	MBC (μg/mL)
*E. coli* ATCC 25922	8	16
*S. aureus* ATCC 25923	8	16
*S. aureus* 1	4	8
*S. aureus* 3	8	16
*P. aeruginosa* PAO1	16	32
*A. baumannii* 34230	4	8
*A. baumannii* 57190	4	8
*K. pneumoniae* 53735	64	128

**Table 2:** Bacterial Biofilm Production Capacity.

Strain	Biofilm Formation
*E. coli* ATCC 25922	Moderate
*S. aureus* ATCC 25923	Strong
*S. aureus* 1	Strong
*S. aureus* 3	Moderate
*P. aeruginosa* PAO1	Strong
*A. baumannii* 3423	Moderate
*A. baumannii* 57190	Moderate
*K. pneumoniae* 53735	Strong

## Data Availability

Data are contained within the article and Supplementary Materials.

## Supplementary Materials

The following supporting information can be downloaded at: https://www.mdpi.com/article/10.3390/molecules29030558/s1, Table S1: the area ratio of membrane permeability; Table S2: the area ratio of ROS; Table S3: docking dynamics simulation RMSD of the protein–peptide; Table S4: docking dynamics simulation RMSF of the protein–peptide.

## Abbreviations

MIC, Minimum inhibitory concentrations; MBC, minimum bactericidal concentration; SEM, Scan electron microscope; CLSM, Confocal laser scanning microscope; LB broth, Luria-Bertani broth; CFU, Colony-Forming Units; *P. aeruginosa* PAO1, *Pseudomonas aeruginosa* PAO1; *E. coli*, *Escherichia coli*; *S. aureus*, *Staphylococcus aureus*; PI, propidium iodide; *A. baumannii*, *Acinetobacter baumannii*; *K. pneumoniae*, *Klebsiella pneumoniae*; EPS, extracellular polysaccharide; CV, crystal violet; QS, quorum sensing. PC, positive control.
